# Small cell lung cancer with small intestinal metastasis: Case report and literature review

**DOI:** 10.1515/biol-2025-1073

**Published:** 2025-04-29

**Authors:** Hai Zeng, Ziguo Liu, Min Zhu, Tian-e Zhang

**Affiliations:** Department of Oncology, The First Affiliated Hospital of Yangtze University, Jingzhou, 434020, China; Department of Oncology, Dazhu County People’s Hospital, Dazhou, 635000, China; The First Affiliated Hospital of Yangtze University, Jingzhou, 434020, China

**Keywords:** lung cancer, gastrointestinal metastasis, small cell lung cancer, small intestinal metastasis, gastrointestinal perforation

## Abstract

Patients with small cell lung cancer (SCLC) typically exhibit a poor prognosis, often receiving diagnoses at an advanced stage. Despite recent advances in immunotherapy, the median survival remains approximately one year. Gastrointestinal metastases from lung cancer, based on clinical experience, are exceedingly rare and associated with dire prognoses. This article details the diagnosis and management of an unusual case of SCLC with gastrointestinal metastasis. The patient’s survival was notably prolonged compared to typical SCLC outcomes, providing significant clinical insight.

## Graphical abstract

**Figure:**
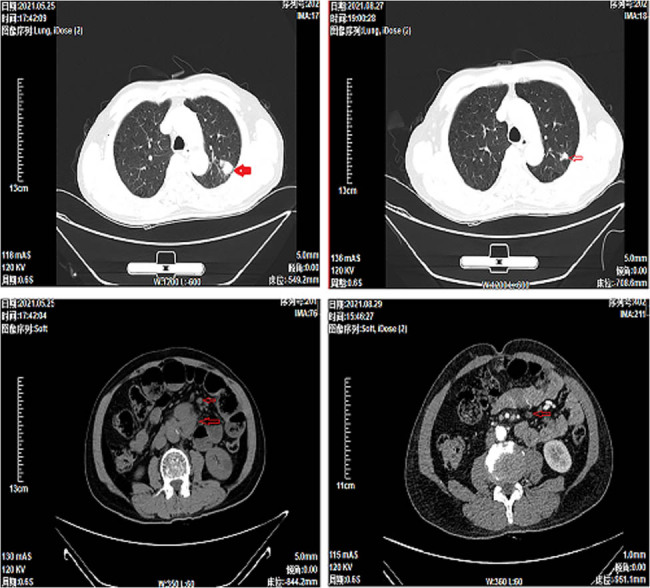

## 1 Introduction

Lung cancer is primarily categorized into two types: non-small cell lung carcinoma and small cell lung carcinoma (SCLC). SCLC, a poorly differentiated neuroendocrine tumor, accounts for approximately 15% of all lung cancer cases [1]. It is characterized by rapid proliferation, early metastasis, and a poor prognosis. Common metastasis sites for SCLC include the liver, adrenal glands, bone, and brain, with gastrointestinal metastases being particularly uncommon [2,3,4,5]. Many patients with gastrointestinal metastases from lung cancer remain asymptomatic, often missing the critical window for effective treatment and thus experiencing disease progression. Clinical detection rates for gastrointestinal metastasis from lung cancer are below 2%, while autopsy rates can reach as high as 11%.

A high index of suspicion is therefore essential for any gastrointestinal symptoms in patients with SCLC. Diagnostic procedures, such as abdominal CT, endoscopy, and Positron emission tomography–computed tomography (PET–CT), should be conducted promptly to detect digestive tract metastases, facilitating early intervention. Treatment strategies incorporating surgery, chemotherapy, and radiotherapy can mitigate symptoms, prevent complications like intestinal obstruction and perforation, and consequently improve both quality of life and prognosis [6,7,8,9,10]. This article presents a rare instance of SCLC metastasizing to the small intestine (ileum), where the patient suffered from intestinal perforation and diffuse peritonitis, among other complications. Aggressive surgical management and standard chemotherapy enabled a survival period exceeding 41 months, aiming to broaden clinicians’ understanding of this malignancy.

## 2 Methods

Following approval from our hospital’s ethics committee, informed consent was obtained from the patient. Previous inpatient medical records, including CT images and pathology slides, were reviewed. Literature searches were conducted using PubMed, CNKI, and MedReading with themes including “small cell lung cancer/gastrointestinal metastasis” and “small cell lung cancer/small intestinal metastasis.” Relevant case reports, literature reviews, and analyses were identified and collected.


**Informed consent:** Informed consent has been obtained from all individuals included in this study.
**Ethical approval:** The research related to human use has been complied with all the relevant national regulations and institutional policies and in accordance with the tenets of the Helsinki Declaration and has been approved by the authors’ institutional review board or equivalent committee.

## 3 Case report

**Figure 1:**
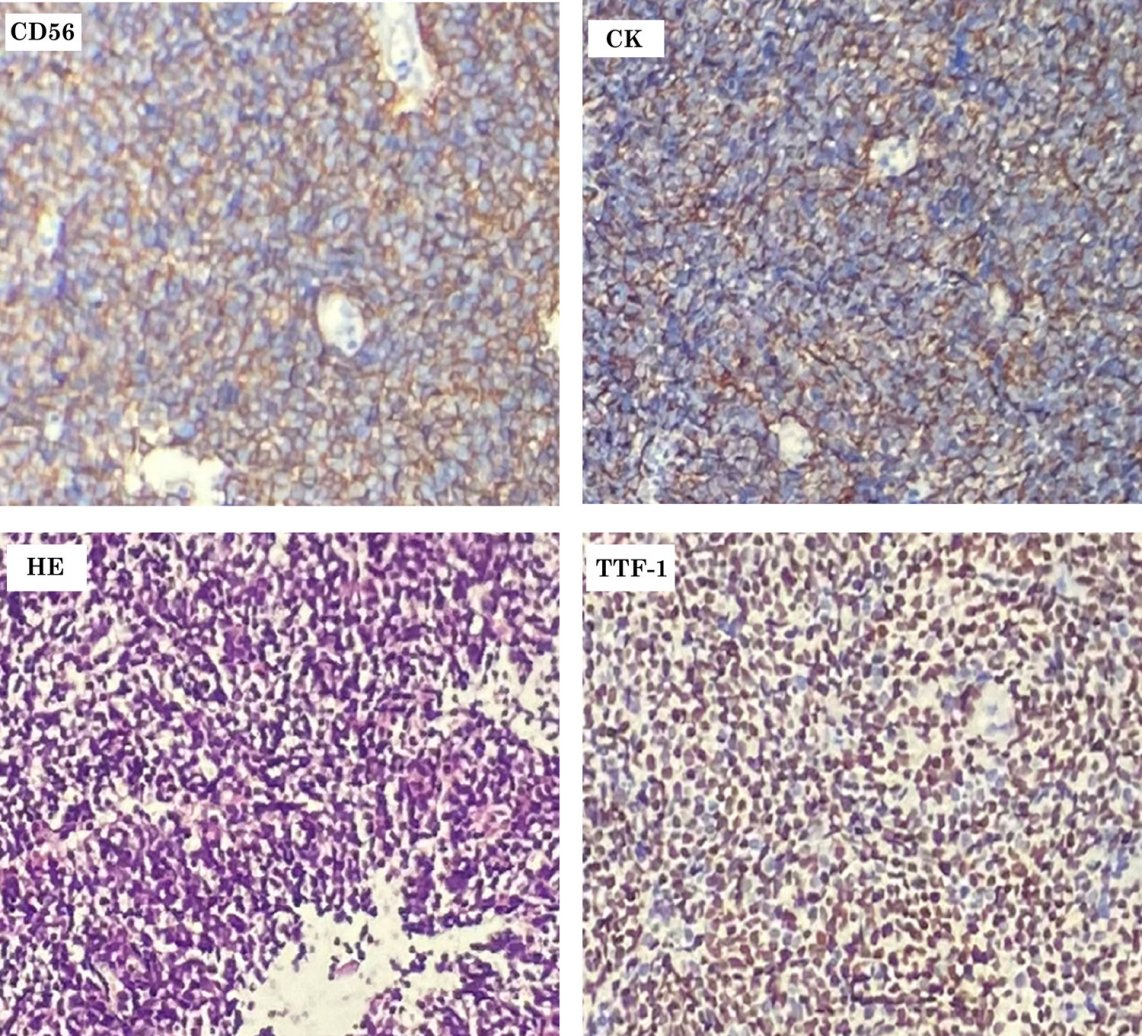
Pathological image.

The patient, a 58-year-old man, reported a 40 pack-year history of heavy smoking and regular alcohol consumption. In April 2021, he sought evaluation for recurrent lower abdominal pain persisting for 6 months at an external hospital, where a gastrointestinal color Doppler ultrasound and abdominal CT were performed. These examinations suggested a space-occupying lesion in the small intestine, indicative of a potential malignant tumor. A subsequent contrast-enhanced abdominal CT at a tertiary (3A) hospital in Chongqing showed thickening of the small intestinal wall in the left mid-to-lower abdomen, accompanied by mass formation and multiple surrounding lesions, suggestive of small intestinal malignancy with lymph node metastasis and possible pelvic peritoneal involvement. The patient exhibited no respiratory symptoms such as cough, sputum production, or shortness of breath. A comprehensive chest CT included in the routine workup identified a space-occupying lesion in the apical-posterior segment of the left upper lobe, highly indicative of lung cancer, with enlarged mediastinal lymph nodes (stations 5 and 10L), suggesting potential metastasis. Needle biopsies were performed on the lung, mediastinal lymph nodes, and the small intestinal mass. All three lesions demonstrated features of high-grade neuroendocrine carcinoma, with suspected small-cell carcinoma based on morphological and immunohistochemical (IHC) findings (Figure 1). IHC results were as follows: CK (+), CD56 (+), CgA (−), Syn (focal +), TTF-1 (+), P63 (−), Vimentin (−), WT-1 (−), Desmin (−), CD99 (+), NSE (−), and Ki-67 (70% positivity).

**Figure 2:**
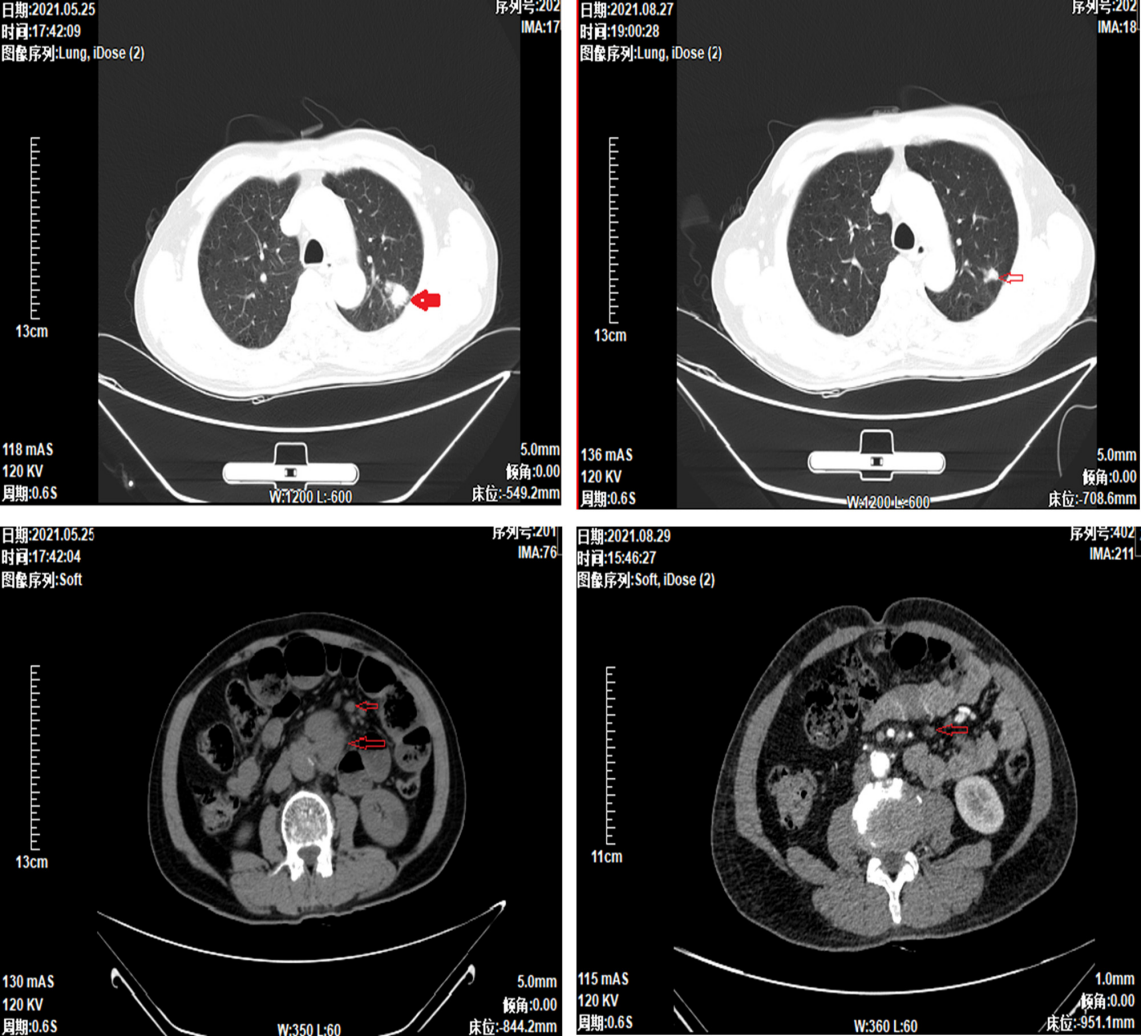
Comparison of lung and small intestinal tumors after the third chemotherapy (August 2021) with those after the first chemotherapy (May 2021), showing significant reduction in size.

In summary, the patient was diagnosed with extensive-stage SCLC and was not eligible for surgical or curative radiotherapy. In May 2021, at a tertiary hospital in Chongqing, three cycles of IC chemotherapy (carboplatin 450 mg + irinotecan 240 mg) were administered. The patient developed incomplete intestinal obstruction following the first and second chemotherapy cycles, which improved with conservative treatment. Relief of abdominal pain was noted after two chemotherapy cycles. Following the third cycle, a follow-up examination, including a CT scan of the chest and abdomen, was conducted at our hospital. The CT scan showed reductions in the size of the lesions in the left lung, mediastinal lymph nodes, and small intestine (Figure 2).

**Figure 3:**
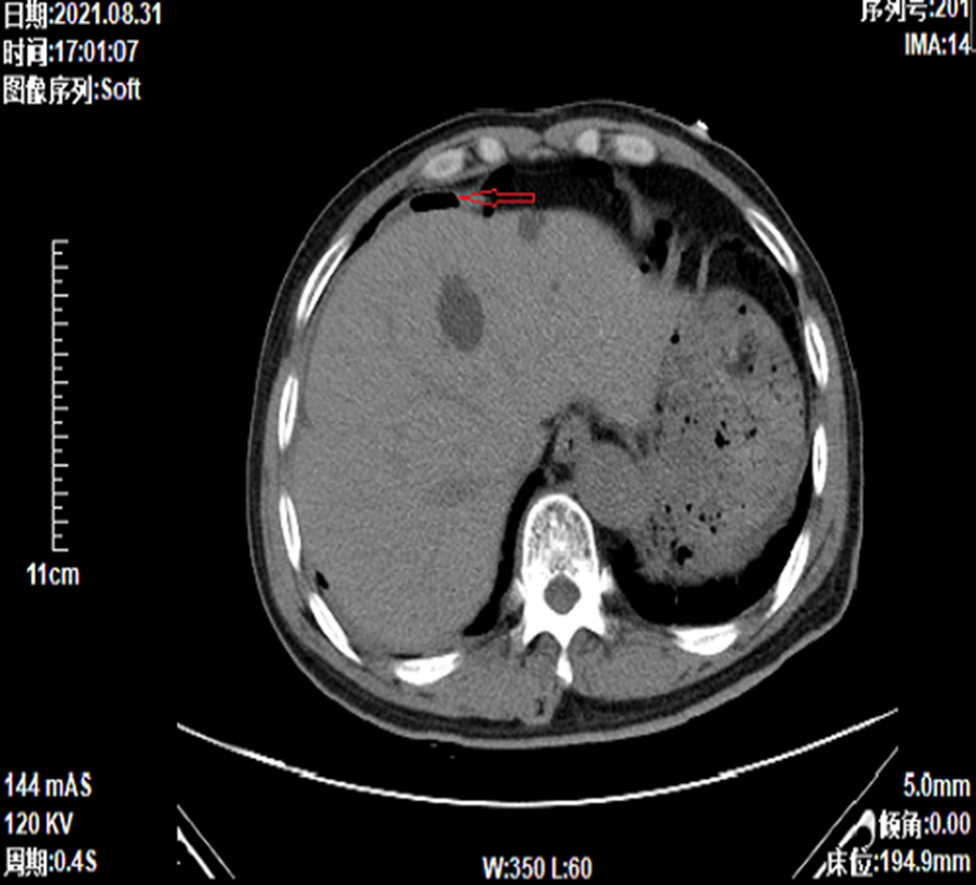
Subphrenic free gas.

In August 2021, after the third chemotherapy cycle, the patient sought medical advice due to a fever but did not report symptoms such as sore throat, cough, abdominal pain, diarrhea, urinary urgency, or dysuria. Blood cultures were immediately obtained upon admission and returned negative 3 days later. A complete blood count showed leukopenia with a white blood cell count of 3.37 × 10^9^/L, an elevated C-reactive protein level of 63.34 mg/L, and treatment with broad-spectrum antibiotic piperacillin/tazobactam at a dose of 4.5 g twice daily was initiated. On the third day, an enhanced CT scan of the abdomen was performed again, revealing a small intestinal mass and enlarged peripheral lymph nodes. On day 5, the patient experienced severe abdominal pain, and a follow-up abdominal CT scan showed gastrointestinal perforation (Figure 3) and diffuse peritonitis.

**Figure 4:**
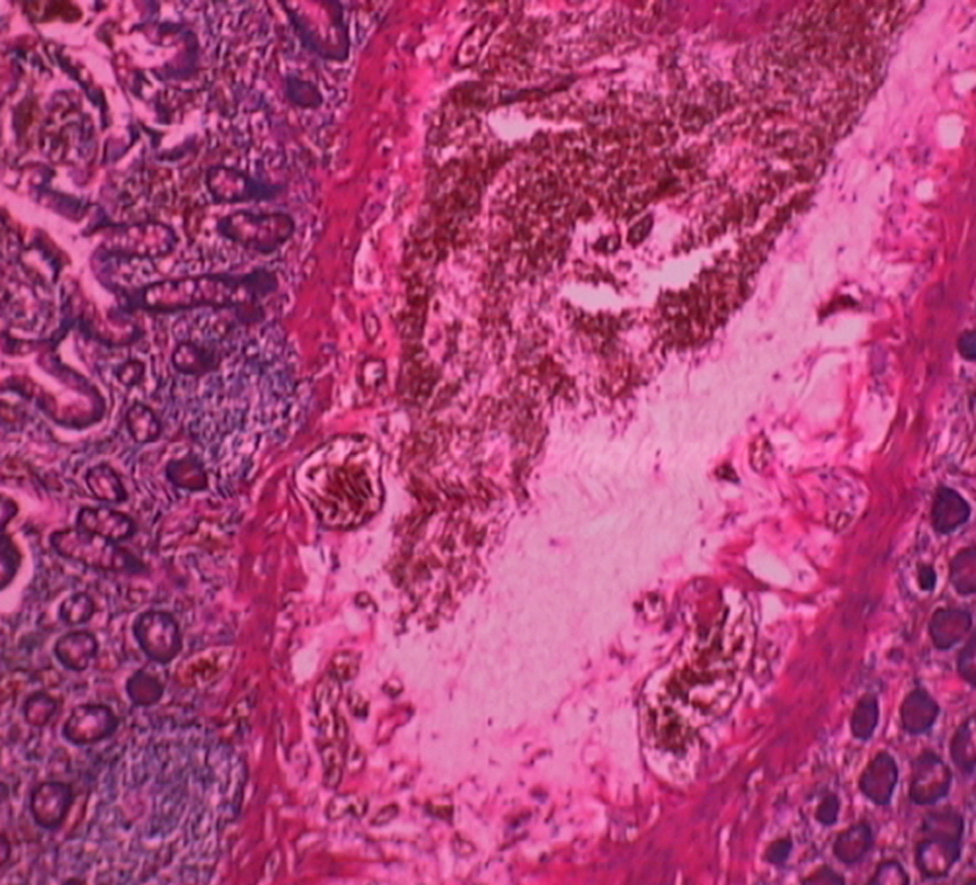
Postoperative pathological image.

The patient was urgently transferred to the gastrointestinal surgery department for an exploratory laparotomy, ileal resection, and anastomosis. During the operation, approximately 500 mL of fecal ascites was found in the abdomen and pelvis. A hard mass, approximately 3 cm × 2 cm, was located in the small intestine, approximately 150 cm proximal to the cecum. A perforation with a diameter of about 3 cm was observed at the mesenteric border, resulting in leakage of small intestinal contents. Postoperative pathology confirmed intestinal necrosis (Figure 4). After the surgery, the abdominal mass was no longer detectable.

In October 2021, more than a month after the initial operation, the patient had recovered well and underwent another cycle of IC chemotherapy at a Grade 3A hospital in Chongqing. In November 2021, he returned to our department for additional follow-up IC chemotherapy (carboplatin 600 mg + irinotecan 200 mg), completing a total of five cycles. The patient opted out of prophylactic brain radiation therapy. Follow-up evaluations were scheduled every 3 months, during which stability of the lung and abdominal lymph node lesions was confirmed, and regular follow-ups are ongoing.

## 4 Discussion

The median overall survival (OS) for patients with extensive-stage SCLC is estimated at 10–12 months, while it extends to approximately 17 months for those with limited-stage disease [11]. Research suggests that survival for patients with small intestinal metastasis and perforation secondary to lung cancer typically does not exceed 16 weeks [12]. The average duration from diagnosis to death for patients with lung cancer metastases to the upper gastrointestinal tract is 5.5 months [13]. Chemotherapy for patients with unresectable gastrointestinal metastases has reportedly extended survival to 23 weeks in certain cases [14]. A delayed diagnosis of gastrointestinal metastases poses a fatal risk due to potentially severe complications such as intussusception, intestinal perforation, intestinal obstruction, and bleeding.

Using the literature search tools previously mentioned, it was discovered that cases of SCLC metastasizing to the gastrointestinal tract are exceptionally rare. This rarity is attributed to the low clinical detection rate of such metastases; more often, they are identified through large-scale autopsy studies [2,4,5,12,15,16,17]. Studies indicate that the clinical incidence of gastrointestinal metastases in lung cancer patients is less than 2%, whereas in autopsy studies, this incidence may rise to as high as 11% [6,7]. The primary reason for this discrepancy is the asymptomatic nature of most patients with gastrointestinal metastases [16].

A case reported in Japan in 2006 involved a patient diagnosed with extensive-stage small cell carcinoma of the right lung who received chemotherapy. During chemotherapy preparation, the patient experienced a decrease in hemoglobin levels (from 11.2 to 8.2 g/dL) and tested positive on a fecal occult blood test. An enhanced abdominal CT scan indicated a jejunal mass. Surgical resection was performed, and a postoperative biopsy confirmed metastatic lesions from SCLC. This case later developed colonic metastasis, leading to ileus, deterioration of the patient’s condition, and death 3 years post-surgery [6].

Lv et al. [8] reported a case in 2010 of SCLC with colonic metastasis and intestinal perforation. The patient was treated with the EP regimen and thoracic radiotherapy upon diagnosis. About 9 months post-treatment, she developed persistent left lower abdominal pain, which worsened after 3 months, accompanied by symptoms indicative of intestinal obstruction, such as abdominal distension, cessation of defecation and flatus passage, and vomiting. Contrast-enhanced CT imaging revealed multiple liver metastases and localized thickening of the descending colon. Palliative surgical resection of the colonic lesion was performed; intraoperatively, colonic perforation was discovered, and the postoperative diagnosis confirmed colonic metastasis from SCLC. Three months post-surgery, patient follow-ups were ongoing, with no specific survival periods documented.

Di et al. [18] retrospectively studied 100 cases of lung cancer with intestinal metastasis in 2014, which included 5 cases of SCLC. The study noted that the OS of lung cancer patients with intestinal metastases was poor, averaging 2.3 months.

Costa Almeida et al. [14] reported a case in 2015 of SCLC with gastric metastasis. Following the diagnosis of limited-stage disease, the patient underwent chemoradiotherapy and achieved a complete response. However, the patient later presented with abdominal pain, and an upper abdominal CT scan revealed abnormal thickening of the gastric wall and a space-occupying lesion in the liver-stomach space. A biopsy confirmed metastases to the stomach, and the patient died 3 months later.

Tanriverdi et al. [2] reported a 2014 case, published in 2020, of SCLC with metastases to the duodenum and descending colon, diagnosed in Turkey. The patient, presenting with abdominal pain, rectal bleeding, and shortness of breath, underwent CT and gastrointestinal endoscopy, which revealed masses in the lungs, duodenum, and descending colon. The final diagnosis was SCLC with metastases to the duodenum, descending colon, and brain. Palliative chemotherapy and radiotherapy for brain metastases were administered, but the patient succumbed to the disease 5 months following the diagnosis.

In 2023, Suto et al. [4] reported an asymptomatic case where a cecal mass was unexpectedly discovered during a follow-up examination after cecal polyp surgery, subsequently diagnosed as extensive metastasis of SCLC. Treviño-Arizmendi et al. [17] reported a case where abdominal pain was the initial symptom. Emergency surgery, performed under the assumption of acute appendicitis, led to a postoperative pathological diagnosis of small cell neuroendocrine carcinoma. Based on the pathological results from the lung, the origin was determined to be pulmonary. No final survival time was documented for these cases of unexpected diagnoses of SCLC with gastrointestinal metastases.

Abdominal pain is the most common symptom in patients with lung cancer metastasis to the digestive tract, occurring in up to 80% of cases [16]. In the cases reviewed, abdominal pain was a frequent symptom. Other symptoms reported include nausea, vomiting, melena, weight loss, constipation, hematemesis, anemia, and blood in the stool [2,3]. In instances of hematemesis, hematochezia, and melena, clinicians often consider endoscopy as a diagnostic tool. However, other symptoms might be dismissed as common digestive issues and overlooked. Thus, it is essential to monitor all suspicious gastrointestinal symptoms closely throughout the disease course and to conduct necessary examinations promptly.

Emergency surgery may act as a positive prognostic factor for patients with primary lung cancer and gastrointestinal metastases, provided the patient’s overall condition permits [5,6,7,14]. However, it is important to recognize that some authors argue that surgery may only relieve symptoms associated with gastrointestinal metastases, without necessarily improving overall prognosis or extending survival, with post-surgical survival often not exceeding 16 weeks [8,12].

Why do these divergent views exist? Insights arise from two mechanisms leading to intestinal perforation. Yang et al. [12] observed that small bowel involvement often results in complications such as perforation, obstruction, or bleeding, with intestinal perforation being the most common. They suggest that tumor invasion into the intestinal wall, either partially or wholly, increases the likelihood of necrotic tumor perforation. Addressing intestinal perforation surgically at the perforation site alone, without systemic tumor control, is unlikely to improve prognosis. Several studies have indicated that chemotherapy can also lead to intestinal perforation [7,13,14]. Assuming effective systemic treatment, emergency surgery to manage intestinal perforation can be life-saving. The cases reported in Japan in 2006 [6] and those in our report were successfully treated surgically, resulting in a favorable prognosis. This outcome relates to intestinal perforation due to tumor regression following chemotherapy.

## 5 Conclusion

Abdominal pain is the most common symptom of digestive tract metastasis from lung cancer, with other symptoms including nausea, vomiting, melena, hematochezia, and anemia. Attention is urgently required for patients with SCLC and primary lung cancer who exhibit these gastrointestinal symptoms. It is crucial to analyze the causes of these symptoms and enhance diagnostic evaluations through abdominal CT, endoscopy, PET–CT, and other relevant examinations. When metastatic lesions are identified, and the patient’s overall health and disease stability permit, a more aggressive surgical approach is recommended. Due to the limited sample size in existing studies, a need for larger-scale studies to substantiate the effectiveness of surgical interventions is evident.
